# Influence of Age, Sex, Vascular and Lifestyle Factors on Alzheimer's Disease biomarkers among Indigenous Africans: Preliminary Findings from the VALIANT Cohort

**DOI:** 10.1002/alz70856_106884

**Published:** 2026-01-08

**Authors:** Tolulope O Akinyemi, Andrea L. Benedet, Oladotun V Olalusi, Gabriel O Ogunde, Joseph O Yaria, Eniola O Cadmus, Femi O Popoola, Mayowa Ogunronbi, Dorcas Olujobi, Olaoluwa Famuyiwa, Joshua O Akinyemi, Mayowa O Owolabi, Roman Romero‐Ortuno, Adesola Ogunniyi, Margaret Pericak‐Vance, Raj Kalaria, Chinedu T Udeh‐Momoh, Henrik Zetterberg, Rufus O. Akinyemi

**Affiliations:** ^1^ Lead City University, Ibadan, Oyo, Nigeria; ^2^ Department of Psychiatry and Neurochemistry, Institute of Neuroscience and Physiology, The Sahlgrenska Academy, University of Gothenburg, Mölndal, Sweden; ^3^ Department of Neurology, University College Hospital, Ibadan, Oyo, Nigeria; ^4^ Neuroscience and ageing research unit, Institute of Advanced Medical Research and Training (IAMRAT), College of Medicine, University of Ibadan, Ibadan Nigeria, Oyo, Nigeria; ^5^ John P. Hussman Institute for Human Genomics, University of Miami, Miami, FL, USA; ^6^ Institute for Advanced Medical Research and Training, College of Medicine, University of Ibadan, Ibadan, Oyo, Nigeria; ^7^ University College Hospital, Ibadan, Oyo, Nigeria; ^8^ University of Ibadan and University College Hospital, Ibadan, Oyo, Nigeria; ^9^ University of Ibadan College of Medicine, Ibadan, Oyo, Nigeria; ^10^ College of Medicine, University of Ibadan, Ibadan, Oyo, Nigeria; ^11^ Institute of Advanced Medical Research and Training (IAMRAT), College of Medicine, University of Ibadan, Ibadan Nigeria, Oyo, Nigeria; ^12^ Neuroscience And Ageing Research Unit, Institute for Advanced Medical Research and Training, College of Medicine, University of Ibadan, Ibadan, Nigeria; ^13^ College of Medicine, University of Ibadan, Ibadan, Oyo State, Nigeria; ^14^ Trinity College Institute of Neuroscience, Dublin, Ireland; ^15^ Global Brain Health Institute, Trinity College, Dublin, Ireland; ^16^ Global Brain Health Institute, Dublin, Ireland; ^17^ Global Brain Health Institute (GBHI), Trinity College Dublin, Dublin, Ireland; ^18^ Neuroscience and Aging Research Unit, Institute of Advanced Medical Research and Training, College of Medicine, University of Ibadan, Ibadan, Nigeria, Ibadan, Oyo, Nigeria; ^19^ Department of Neurology, University of Miami Miller School of Medicine, Miami, FL, USA; ^20^ University of Miami, Miami, FL, USA; ^21^ Institute for Ageing and Health, Newcastle University, Newcastle, United Kingdom; ^22^ Wake Forest University School of Medicine, Winston‐Salem, NC, USA; ^23^ Brain and Mind Institute, Aga Khan University, Nairobi Province, Nairobi, Kenya; ^24^ University of Gothenburg, Mölndal, Sweden; ^25^ Sahlgrenska University Hospital, Mölndal, Sweden; ^26^ Clinical Neurochemistry Laboratory, Sahlgrenska University Hospital, Mölndal, Västra Götalands län, Sweden; ^27^ Department of Neurodegenerative Disease, UCL Institute of Neurology, London, United Kingdom; ^28^ Institute of Neuroscience and Physiology, Sahlgrenska Academy at the University of Gothenburg, Gothenburg, Sweden; ^29^ Institute of Neuroscience and Physiology, University of Gothenburg, Gothenburg, Mölndal, Sweden; ^30^ Institute of Neuroscience and Physiology, the Sahlgrenska Academy at the University of Gothenburg, Gothenburg, Sweden; ^31^ Institute of Advanced Medical Research and Training, College of Medicine, University of Ibadan, Ibadan, Oyo State, Nigeria; ^32^ Africa Dementia Consortium, Ibadan, Ibadan, Nigeria

## Abstract

**Background:**

The neuropathological characterization of Alzheimer disease and related dementias (ADRD) has expanded globally through biofluid‐based biomarker research, with increasing focus on less invasive novel plasma biomarkers. However, data on ADRD biomarkers among indigenous Africans(IA) remain limited. Further, the influence of social determinants of health (SDOH), vascular risk factors(VRFs), and lifestyle determinants on ADRD biomarkers among Africans is unclear. To address this, we investigated plasma concentrations of five key ADRD biomarkers in a large community‐based cohort of older Nigerians, examining their associations with lifestyle factors, SDOH and VRFs.

**Method:**

The Vascular heAlth, fraiLty, and cognItion in Ageing Nigerians (VALIANT) is a longitudinal community‐based study conducted in Ibadan Northeast Local Government Area, Oyo State, Nigeria. A total of 1,036 participants were recruited through multi‐stage, stratified cluster random sampling method, and underwent cognitive and functional assessments. Plasma concentrations of AD biomarkers (Aβ40, Aβ42, NfL, GFAP and *p*‐Tau 217) were measured with single‐molecule‐assay (SIMOA) technology, and the data square‐root transformed. Multiple linear regression models assessed the associations between biomarker levels and 18 independent covariates, including age, sex, VRFs, diet, physical strength, and social network scale. *p*‐value <0.05 was deemed statistically significant.

**Result:**

AD biomarker data were available for 1026 participants (age range: 50‐107 yrs; mean age: 65+/‐10.8yrs;27.2% male). At baseline, 3.4% of participants had dementia, and 11% had mild cognitive impairment (MCI), while the majority were cognitively unimpaired. All ADRD biomarkers varied significantly by age, sex (higher in males) and hand grip strength. Notably, Aβ40 and GFAP were independently influenced by social network scale, suggesting potential social determinants of amyloid deposition and astrocytic activation. Diabetes mellitus and regular intake of green leafy vegetables significantly impacted NfL levels, indicating metabolic contributions to axonal degeneration. Dyslipidemia, BMI, and educational attainment were independently associated with GFAP expression, highlighting metabolic and cognitive influences on neuroinflammatory pathways.

**Conclusion:**

This study provides novel insights into how sociodemographic factors, diet, VRFs, and SDOH influence AD biomarker expression in IAs. The observed associations with amyloid deposition (Aβ40), neuroinflammation (GFAP), and axonal integrity (NfL) highlight distinct mechanistic pathways that warrant further investigation. Future work will evaluate associations with cognitive and clinical outcomes.

